# Horizontal Hostility among Non-Meat Eaters

**DOI:** 10.1371/journal.pone.0096457

**Published:** 2014-05-08

**Authors:** Hank Rothgerber

**Affiliations:** Department of Psychology, Bellarmine University, Louisville, Kentucky, United States of America; The University of New South Wales, Australia

## Abstract

The present study examined intergroup judgments made between four groups of non-meat eaters: health vegetarians; ethical vegetarians; health vegans, and ethical vegans. Consistent with hypotheses based on horizontal hostility and the need to maintain ingroup distinctiveness, ethical vegetarians gave unfavorable evaluations to health vegetarians relative to vegans, especially when the mainstream omnivore group was made salient. Contrary to expectations, vegans gave relatively more favorable evaluations to ethical vegetarians than health vegetarians when mainstream salience was low. This was especially true for vegans who were motivated primarily by ethical concerns. When mainstream salience was high, vegans did not distinguish between the vegetarian subgroups. Results suggest that one’s motives for abstaining from meat often play a larger role in this type of intergroup perceptions than one’s dietary practices.

## Introduction

Despite increasing claims the last decade that meat consumption harms the environment, personal and public health, and animals [1], vegetarianism still remains a relatively infrequent practice in the U.S., accounting for 3% of the population [2]. Estimates in other western nations are generally low as well, ranging from 9% in Germany to less than 2% in Denmark and France [3].

Despite being relatively small, there is quite a bit of dietary variation within this minority population. Divisions based on diet include: semi-vegetarians, which include pescetarians and pollotarians; ovo, lacto, and ovo-lacto vegetarians; vegans; and fruitarians. The present work is concerned with how favorably non-meat eating subgroups perceive each another. Theoretically, this provides an opportunity to test a framework explicating relations between multiple minority groups. From a practical perspective, for those viewing meat eating as a social problem, it may be helpful to understand the dynamics between these groups, such as how unified they are, as consistency is critical in influencing the majority [4].

One initial challenge for investigators pursuing these perceptions is in how to define and distinguish between the numerous types of non-meat eaters. Even in something as straightforward as defining vegetarianism, there is inconsistency in the literature and in how people self-identify [5]. Although the focus is often on specific dietary practices, individuals also differ in their *motives* for abstaining from meat [5] [6], with the two most common motives involving ethics (primarily the treatment of animals but also the environment) and personal health. In this study, diet and motives were combined to create three target groups: *health vegetarians* (those who abstain from eating meat and seafood for reasons of personal health), *ethical vegetarians* (those who abstain from eating meat and seafood for reasons of harm to animals and/or the environment), and *vegans* (those who avoid all animal products including dairy and eggs). It was believed that this was the simplest and most meaningful way to reduce non-meat eaters into manageable and recognizable categories. Rothgerber [6], for example, found this distinction helpful in explaining how vegetarian pet owners handled the psychological consequences of living with meat-eating pets. Vegans as targets were not divided according to their motives because past studied have lumped them together, likely because of their smaller size or because the extreme nature of their diet is highly salient. In the analysis though, vegans as participants were divided into health and ethical types.

To date, there appear to be no published studies that examine all these groups simultaneously and the perceptions that they have of each other. Several studies have compared two of these groups and revealed general patterns of ingroup bias and outgroup ambivalence. For example, Povey and colleagues [7] found that vegetarians described veganism as humane, healthy, and ethical but primarily, restrictive. Vegans perceived vegetarians as humane and healthy on the one hand, but the most salient belief was that vegetarians were hypocritical. The study did not ask vegans to distinguish between health and ethical vegetarians, potentially obscuring important differences nor did it assess whether vegans differed in their perceptions depending on their own motives. It also did not distinguish between vegetarian subgroups in their perceptions of vegans. In an online qualitative study, ethical vegetarians were critical of health vegetarians whom they perceived as selfish and insufficiently radical [8]. There was some common ground though as both groups seemed to become more environmentally-committed over time. It is unclear how many of the 33 participants were vegan though, and whether vegans differed systematically from vegetarians in their perceptions and in how they were perceived.

### Horizontal Hostility

To account for these piecemeal findings and offer a theoretical rationale for explaining perceptions between multiple non-meat eating groups, the present work adopted a framework by White, Langer and colleagues [9] [10] that has been useful in understanding relations and predicting attitudes between similar minority groups: *horizontal hostility*. Two attributes are relevant to understanding horizontal hostility: similarity, the degree to which groups share relevant characteristics; and distinctiveness, meaning a lack of similarity/having a separate ingroup identity from an outgroup and also the degree to which groups differ from the mainstream. Although the majority may expect members of similar minority groups to be favorably disposed toward each other [see 11], a host of studies have revealed that intergroup similarity can either facilitate attraction or hostility [12]. According to horizontal hostility, the direction of similarity – whether the similar outgroup is more mainstream/less distinct (i.e., closer to the majority group) or more extreme/more distinct (i.e., further from the majority group) than the ingroup – moderates the relationship between intergroup similarity and attitudes.

Specifically, minority groups should display relatively unfavorable attitudes toward members of an outgroup that is similar to but more mainstream than the ingroup because such an outgroup poses a unique threat to the distinctiveness of the minority ingroup identity. This follows from the “reactive distinctiveness hypothesis” (see [13]) of social identity theory [14], that threats to intergroup distinctiveness (i.e., too much similarity) would instigate attempts to restore distinctiveness. Similar, less distinctive groups are targets for horizontal hostility because outsiders may lump them together with the minority ingroup and that would threaten the minority group’s distinctiveness in two ways: The minority ingroup boundaries would become unclear and permeable; and the minority ingroup would become larger (and less distinctive) and add members who are closer to the mainstream than the typical group member [9].

White and colleagues [10] generated three hypotheses based on horizontal hostility: (1) evaluations of a similar but less distinct outgroup would be negative relative to evaluations of a similar but more distinct outgroup; (2) evaluations of an outgroup similar to but less distinct than the ingroup would be more negative than evaluations of an outgroup dissimilar and even less distinct than the ingroup; and (3) evaluations of a similar but less distinct outgroup would be more negative when the mainstream majority was highly salient. The rationale for this last hypothesis is that when the mainstream majority group is a salient part of the intergroup context, the dimension that positively distinguishes the minority group from the majority becomes valued and meaningful. Thus, in such contexts the motivation to achieve and maintain ingroup distinctiveness promotes hostility toward a similar but less distinct minority group and more positive evaluations of a similar but more distinct minority group.

The asymmetric pattern of judgments implied in these predictions has been demonstrated in various types of groups that can be ordered along a mainstream-extreme continuum, including Jewish congregations, minority Greek political parties, and soccer teams [9] [10]. Of considerable interest for the present research, in one study 76 vegetarians and 37 vegans rated the ingroup and outgroup in a context that either made the mainstream omnivore group highly salient or not [10]. Vegetarians rated vegans more favorably when omnivores were highly salient than when they were not because such affiliation with an extreme group helps differentiate the ingroup from the mainstream. Consistent with hypothesis 3, vegans rated vegetarians more negatively in the high mainstream salience condition than in the low mainstream salience condition. Although this study supports a horizontal hostility framework and its emphasis on protecting a threatened social identity through achieving distinctiveness, for our purposes it fails to distinguish between vegetarian subgroups as targets and participants and between vegan subgroups as participants. This leaves open several questions including whether vegans would evaluate health and ethical vegetarians similarly, whether this would depend on their own diet motives, and whether health and ethical vegetarians would evaluate vegans similarly.

### A Continuum of Non-meat-eating Subgroups

In attempting to order non-meat eating subgroups along a continuum, it is clear that vegans are highly distinct and on the opposite end from omnivores. By adhering to a more restrictive diet, vegans challenge dietary conventions even more than vegetarians. To illustrate their deviation from the majority, vegans offered more reasons for their diet and believed that animals were more similar in their emotionality to humans than did vegetarians [6]. Relative to vegetarians, they have also been found to express greater concern over the impact of their diet on animal welfare and the environment [15], were less likely to feed their pets a meat-based diet [6], and scored higher on Herzog and colleagues’ animal attitudes scale [16] in unpublished data by Ruby, Cheng, & Heine. Suggestive of their radicalism, vegans indicated that their diet is more of a lifestyle than a diet [8], and for some, these convictions run so strong they refuse sexual intimacy with non-vegans [17]. Although plausible given the information that follows, it is unclear whether vegans motivated by ethical concerns would display these tendencies more strongly than vegans motivated by health concerns because prior research has tended to lump all vegans into one category.

In ordering the two vegetarian subgroups along the mainstream-minority continuum, several pieces of evidence converge to suggest that ethical vegetarians are more extreme and distinct than health vegetarians. Because this insight is the basis for subsequent predictions, its rationale warrants further discussion. First, ethical vegetarians and vegans are ideologically similar. That is, vegans are motivated more by ethical rather than health concerns [6]. The ideology of ethical vegetarians is more extreme than that of health vegetarians, framing their diet within a philosophical, ideological, or spiritual context [8] and being more motivated by humanistic values [18]. Ethical vegetarians perceived themselves as more radical than health vegetarians [8]. Indeed, they displayed stronger opposition to foxhunting and capital punishment and greater support for nuclear disarmament initiatives than other vegetarians [19], were more likely than health vegetarians to feed their pet a vegetarian diet and expressed greater concerns over feeding their pet an animal-based diet [6], and compared to health vegetarians reported being more disgusted by meat, showed more concern when they saw others eat meat, expressed stronger emotional reactions to meat consumption, and believed that meat causes undesirable changes in personality [20]. In addition, Rothgerber has found in unpublished data that the responses of ethical vegetarians fall between those of health vegetarians and vegans on several measures: number of reasons offered for their vegetarianism and belief that humans and animals share emotional experiences. Finally, ethical vegetarians were more likely to abruptly adopt their diet and as might be expected given their ideological compatibility, were more likely to transition to veganism than health vegetarians [21].

In contrast, the focus within health vegetarians is internal, addressing desires to sustain good health and avoid illness. Emphasis is placed on personal health, fitness and energy rather than outward toward other living creatures [8]. Rather than being driven by ideology, many health vegetarians traced their diet to personal experiences. Some researchers have suggested that the ideology of health vegetarians is more conservative and normative value driven, concerned with personal safety and security [18]. Health vegetarians tend to gradually eliminate meat from their diet and are less likely to eliminate all animal products from their diet [21]. As such, when considering factors beyond diet, they appear to be closer to the mainstream and less distinct than ethical vegetarians.

In the present study, health vegetarians, ethical vegetarians, health vegans, and ethical vegans rated their own group and the outgroups in a context that either made the mainstream group (omnivores) salient or not. Based on horizontal hostility, the need for a distinct ingroup identity, and on the logic that these minority groups could be arranged along a continuum from omnivores-health vegetarians-ethical vegetarians-vegans, the following hypotheses were made:

*Hypothesis 1*: Ethical vegetarians will give vegans more favorable global evaluations than they will health vegetarians;

*Hypothesis 2*: Vegans will give health vegetarians more favorable global evaluations than they will ethical vegetarians (with it unclear whether vegan subgroups would differ from each other in this assessment); and

*Hypothesis 3*: When the mainstream omnivore majority is a salient part of the comparative context, the effects in the first and second hypotheses will be stronger than when the mainstream majority is not made salient.

## Method

### Ethics Statement

This research was approved by the Bellarmine University Institutional Review Board and met all applicable standards for the ethics of experimentation and research integrity. Written informed consent was obtained from participants before they completed the questionnaire. All participants were 18 years of age or older.

### Participants and Procedure

Participants were recruited primarily through the Vegetarian Resource Group (www.vrg.org). According to their website, “The Vegetarian Resource Group (VRG) is a non-profit organization dedicated to educating the public on vegetarianism and the interrelated issues of health, nutrition, ecology, ethics, and world hunger.” A brief recruitment notice for a study on vegetarians and vegans was posted on the organization’s blog, facebook and twitter accounts, and in national and local newsletters along with a link to the survey monkey website hosting the survey. Participants were offered entry into a $50 lottery drawing in appreciation for their participation. The survey was accessible from October 29, 2012 to November 15, 2012.

During this period, 464 individuals completed all the survey questions. After excluding respondents for reasons stated later, the final dataset included 431 participants. Of the final sample, 83% were females. 81% listed the U.S. as country of origin; 9% listed Australia, 6% Canada, 4% Europe and less than 1% another country. The mean age of participants was 39.0 (SD = 12.21). The sample was well-educated: 4% reported having less than a high school education, 9% had high school or GED, 9% had an associate’s degree, 41% reported having a college degree, and 37% reported having a graduate degree.

The salience of the majority group was manipulated by creating alternative survey versions. In the high mainstream salience condition, respondents were first asked to evaluate omnivores before judging the three non-meat eating minority groups, whereas in the low mainstream salience condition, respondents rated the three minority groups without reference to omnivores. Each participant completed the following measures as part of a larger study.

### Measures

#### Diet

Participants’ diet was assessed with a single-item question asking them to choose which diet applied to them: vegetarian, vegan, or none of the above. Participants who selected the last response (n = 31) were excluded from the analysis.

#### Dietary motives

To assess their motives for following their current diet, participants chose between one of the following options: “I avoid eating meat primarily for ethical reasons; I avoid eating meat primarily for health reasons; Ethical and health reasons are about equal in importance to me; or none of the above.” Participants giving the last response (n = 2) were excluded. Participants choosing health motives or a combination of health and ethical motives were combined into a single category of health vegetarians. There were two reasons for this decision. First, prior research has show that health and mixed-motive vegetarians are similar in their perceptions [6] [22]. Second, there were no differences between these two groups in their evaluations in the present study (e.g., target effect: *F*(2,414) = 1.94, *p* = .144, ηρ^2^ = .01). In total, combining diet and motives, 16% of respondents were considered health vegetarians (n = 69), 21% ethical vegetarians (n = 90), 26% health vegans (n = 113), and 37% ethical vegans (n = 159).

#### Global evaluations

To assess global evaluations, participants were asked to give their opinions about the following groups based on what they eat and why: Personally-Committed Vegetarians (do not eat meat because of health reasons); Morally-Committed Vegetarians (do not eat meat because of animal cruelty/harm to environment); and Vegans (do not consume meat, fish, or dairy products). In the high mainstream salience condition, respondents were first asked to evaluate omnivores before evaluating the non-meat eating groups. For each group, participants were asked to judge their “overall attitude toward this group” and provide their “overall favorability rating” of each group. Answers were scored on a 7-point Likert scale (1 = *extremely negative/unfavorable*; 7 = *extremely positive/favorable*). Correlations between these two measures were high for each of the three target groups (health vegetarians, *r*(430) = .96; ethical vegetarians, *r*(430) = .96; vegans, *r*(430) = .97), so they were combined to form a single measure.

## Results

Data for the present study will be available from the Dryad Digital Repository: http://dx.doi.org/10.5061/dryad.[sn878].

### Global Evaluation – preliminary Analysis

A repeated-measures ANOVA with participants’ group identity (health vegetarian, ethical vegetarian, health vegan, ethical vegan) and mainstream salience (low, high) as between-subjects factors, and the target (health vegetarian, ethical vegetarian, vegan) as a within-subjects factor, revealed overall main effects for target (*F*(2,423) = 71.16, *p* = .001, ηρ^2^ = .14) and mainstream salience (*F*(1,423) = 33.03, *p* = .000, ηρ^2^ = .07). Here, health vegetarians were rated the lowest and groups under high mainstream salience were rated lower. Within participants, there was a significant participants’ group × target interaction (*F*(6,416) = 21.10, *p* = .000, ηρ^2^ = .13) qualified by a significant participants’ group × target × mainstream salience interaction (*F*(6,416) = 2.68, *p* = .014, ηρ^2^ = .02). The two-way interaction is most simply explained by a pattern of ingroup bias whereas the three-way interaction can be accounted for by the results of Hypotheses 3. With low mainstream salience, overall target effects disappeared for health and ethical vegetarian respondents. Means appear in Table 1.

**Table 1:** Vegetarians’ and Vegans’ Ingroup and Outgroup Evaluations under Low and High Mainstream Salience.

Target Evaluations						
	Health Vegetarian		Ethical Vegetarian		Vegan	
Participants	Mean	SD	Mean	SD	Mean	SD
Low mainstream salience						
Health Vegetarians (n = 23)	5.78	1.17	5.78	1.24	5.87	1.25
Ethical Vegetarians (n = 38)	5.45	1.39	5.87	1.14	5.61	1.34
Health Vegans (n = 47)	5.40	1.28	5.62	1.15	6.26	1.03
Ethical Vegans (n = 78)	4.89	1.34	5.63	1.28	6.55	0.93
High mainstream salience						
Health Vegetarians (n = 46)	5.20	1.09	5.09	1.19	5.20	1.24
Ethical Vegetarians (n = 52)	4.38	1.25	5.38	1.24	5.37	1.47
Health Vegans (n = 66)	4.88	1.23	4.80	1.13	5.50	1.37
Ethical Vegans (n = 81)	4.47	1.25	4.86	1.53	6.06	1.30

Note. Higher means represent more favorable evaluations.

### Global Evaluation - horizontal Hostility

Consistent with the first hypothesis, ethical vegetarians evaluated health vegetarians less favorably than vegans, *F*(1,89) = 15.53, *p* = .000, ηρ^2^ = .15. However, contrary to Hypothesis 2, vegans did not rate ethical vegetarians less favorably than health vegetarians. In fact, they rated ethical vegetarians *more* favorably than health vegetarians, *F*(1,271) = 16.25, *p* = .000, ηρ^2^ = .06. This main effect was qualified by a target x vegan type interaction, *F*(1,271) = 11.91, *p* = .001, ηρ^2^ = .04. Ethical vegans rated ethical vegetarians more favorably than they did health vegetarians, *F*(1,158) = 28.24, *p* = .000, ηρ^2^ = .15, but health vegans did not discriminate in their ratings of vegetarians, *F*(1,112) = 0.20, *p* = .659, ηρ^2^ = .00. Consistent with the first part of Hypothesis 3, the interaction between target and mainstream salience was significant for ethical vegetarians, *F*(1,88) = 6.81, *p* = .011, ηρ^2^ = .07. As expected, ethical vegetarians evaluated health vegetarians less favorably than vegans when the mainstream group (omnivores) was highly salient, *F*(1,51) = 19.48, *p* = .000, ηρ^2^ = .28. They did not, however, distinguish between the two groups when the mainstream group was not highly salient, *F*(1,37) = 0.57, *p = .453,* ηρ^2^ = .02. The second part of Hypothesis 3 was not supported. Although the target x mainstream salience interaction for vegan participants was significant, *F*(1,271) = 5.53, *p* = .019, ηρ^2^ = .02, counter to predictions, when the mainstream group was highly salient, vegans only marginally significantly distinguished between health and ethical vegetarians, *F*(1,146) = 2.83, *p* = .095, ηρ^2^ = .02, albeit in the wrong direction. Under conditions of low mainstream salience however, they rated health vegetarians significantly less favorably than ethical vegetarians, *F*(1,124) = 27.75, *p* = .000, ηρ^2^ = .02.

## Discussion

Because non-meat eating minority groups do not seemingly compete with one another over scarce material resources – the majority of Western vegetarians hail from the middle class [23] – rather than opting for a model based on realistic conflict theory, the present research focused on identity threats as the most salient feature underlying this unique case of intergroup perceptions. The hypotheses generated from a horizontal hostility framework were partially successful in predicting evaluations between members of non-meat eating minority groups. This lends some support to the notion that these groups can be ordered in their proximity to omnivores as health vegetarians, ethical vegetarians, and vegans.

Although objectively it may be difficult judge which vegetarian subgroup is more mainstream and less distinct based simply on their diet, the present results imply that psychologically at least, ethical vegetarians perceive themselves as being more extreme than do health vegetarians. Consistent with predictions, ethical vegetarians evaluated health vegetarians less favorably than vegans. On a superficial reading, it may appear strange that ethical vegetarians would elevate a group that follows a less similar diet to their own over one that follows a more similar diet. However, it appears that for ethical vegetarians, vegetarianism is about more than the behavior of avoiding meat, but that one’s motives are of central importance. From prior research, it is clear that the motives of ethical and health vegetarians stand in stark contrast. Ethical vegetarians have stronger animal rights concerns, react with greater disgust to eating meat, transition more rapidly to a vegetarian diet, and most importantly, conceptualize their diet as part of a larger philosophical framework [5] [8] [18] [20]. Their focus is outward on preventing harm to animals and to the environment. Ethical vegetarians may wonder how truly committed to these causes health vegetarians are. Fox and Ward [8] have for example, shown examples in which ethical vegetarians reported perceiving health vegetarians to be selfish, boring, insufficiently radical, and inferior.

For health vegetarians, vegetarianism seems more of a personal choice left up to the individual. The creed seems to hold that there is nothing wrong per se with others eating meat – they may have unique dietary issues to consider or they may be attaining good health through other means such as exercise. Presumably, the most a health vegetarian would hope for is that each omnivore makes an informed decision about their diet and its health implications. When Lindeman and Sirelius [18] described health vegetarianism as having a conservative outlook, they were referring to a decidedly apolitical stance seemingly taken by health vegetarians. Because they lack the belief that meat eating is morally wrong, there is less need to convert omnivores and less need to perceive their dietary status as a social movement.

Health vegetarians, then, threaten to take attention away from and undercut the distinctive message of ethical vegetarians. They may induce omnivores to conclude that vegetarianism, rather than being a political movement, is an individual lifestyle choice, removing the moral implications of their behavior. For example, omnivores may glean from health vegetarians that healthier, organic meat free of pesticides is acceptable. If concerns with being perceived properly by the mainstream are important to ethical vegetarians, it becomes apparent why evaluations of health vegetarians relative to vegans only became more negative when ethical vegetarians were induced to think about the mainstream omnivore group. For in these cases, social identity concerns about being perceived as a distinct group would have become more pressing for ethical vegetarians. Future research building upon this work and other studies of intergroup perceptions between health and ethical vegetarians [8], then, needs to account for the moderating role of mainstream salience.

In contrast and counter to predictions, instead of increasing pressure to achieve distinctiveness by derogating a more similar outgroup (i.e., ethical vegetarians), high mainstream salience did not lead vegans to differentiate between vegetarian subgroups. When vegan participants were made to think about omnivores, they appear to have self-categorized on the basis of diet, producing two effects: Differences in distinctiveness between vegetarian subgroups became minimized as attention was placed upon their similar diet; and ingroup-outgroup differences became more striking leading ratings of *both* health and ethical vegetarians to decrease relative to conditions when the mainstream was not salient, paralleling the findings of White and colleagues [10] of increased ingroup bias under mainstream salience. This supports the reflective distinctiveness hypothesis [13] that intergroup differentiation is more likely to occur when groups are clearly distinct. In many practical examples in which omnivores are present, then, vegans likely attend to dietary differences, expanding intergroup differences while reducing intragroup variability. When eating with omnivores, for example, vegans would be more likely to disparage vegetarians for consuming eggs (which leads to male roosters being killed [24]) and dairy (which indirectly supports the veal industry [24]) because their focus is on their own vegan diet and preserving its positivity and distinctiveness. Thus, research showing that vegans perceive vegetarians as hypocritical [7] may be most applicable when the mainstream is highly salient.

The present results suggest that vegan perceptions of vegetarians depend not only upon mainstream salience, but also on which vegetarian subgroup is being evaluated. Future studies should be careful in generalizing vegan perceptions of vegetarians across all vegetarian subgroups. Contrary to the distinctiveness threats outlined by horizontal hostility, vegans distinguished between vegetarians by rating ethical vegetarians *more* favorably then health vegetarians. That is, unlike the far-from-the-mainstream groups studied by White and colleagues [10], vegans did not evaluate the nearest group (ethical vegetarians) less favorably than the group closest to the mainstream (health vegetarians). This overall effect was qualified by two factors: (1) the aforementioned effect of mainstream salience and (2) vegan type, i.e., health vegans did not differentiate in their evaluations of vegetarians. This latter result suggests that future research should be cautious in lumping all vegans together.

To account for the unexpected main effect showing that vegans give higher ratings to ethical vegetarians than to health vegetarians, it is useful to consider how the non-meat eating minority groups studied here may differ from the groups previously considered. In published research on horizontal hostility, there is typically one major dimension distinguishing between minority groups. Greek political parties differ primarily on their political ideology, and Jewish denominations differ in their religiousness. In the case of those abstaining from meat, there are two conceptually distinct factors distinguishing between groups, their dietary practices and their motivation for following such practices. When the mainstream omnivore group was made highly salient, it appears vegans attended to dietary differences between themselves and others. But when the mainstream group was not salient, it appears *motives* for one’s diet became perceptually important, and the motives of ethical vegetarians were perceived more favorably than those of health vegetarians. This seems largely driven by the fact that ethical vegans appear to have more strongly identified with ethical vegetarians and perceived them more favorably than they did health vegetarians, who pose a greater threat to their own vegan ideology. As noted, vegans motivated by health concerns did not demonstrate this form of ingroup bias based on diet motive, failing to distinguish between ethical and health vegetarians.

These conclusions are limited by the nature of the sample. Participants were recruited primarily through a vegetarian website that predominately attracted vegans and those with ethical motivations for their meat abstention. Relative to others, those reading the website may very well be more committed to the vegetarian cause, derive more of their social identity from it, organize their free time around abstaining from meat, and be more socially connected to other vegetarians. They may have thought more about their own and others’ motives for abstaining from meat and formed stronger opinions about distinctions between non-meat eaters. The study did not focus on individual differences, but it is possible that identity pressures and the need to perceive one’s own group in positive and distinct terms may have been stronger among these participants. For vegetarians and vegans who derive less of their identity from their meat eating status, the differentiation between groups observed in the present study may be more muted. Of course, it is also possible that those presumably more identified with the cause may discriminate less between subgroups in an effort to strengthen unity. Future research with other samples may help resolve which possibility is more likely.

In conclusion, the present research adds to a growing body of literature [8] [10] that despite their shared status as non-meat eating minorities, vegetarians and vegans do make evaluative distinctions between each other. Overall, ethical vegetarians evaluated themselves less similarly to health vegetarians than they did vegans, a group they may assume holds a similar worldview, while ethical vegans in turn rated ethical vegetarians closer to the ingroup than they rated health vegetarians. Health vegetarians were similar to the other groups in exhibiting ingroup bias, but they showed little differentiation between ethical vegetarians and vegans. While a bit of an oversimplification, it appears, then, that what is most important among non-meat eating minorities – at least in terms of perceptions of others – is one’s worldview and philosophical framework. The specific diet chosen to embody one’s beliefs seems less critical in these intergroup perceptions. It is understandable that outsiders would focus on the observable behavior of what individuals consume and incorrectly assume that diet is the most important dimension of their non-omnivore status; after all, they likely do not have access to the interior motives of others. However, to those abstaining from meat, the internal motives may constitute a much larger basis of self-definition and in defining others.
